# Snacking on Almonds Lowers Glycaemia and Energy Intake Compared to a Popular High-Carbohydrate Snack Food: An Acute Randomised Crossover Study

**DOI:** 10.3390/ijerph182010989

**Published:** 2021-10-19

**Authors:** Rachel Brown, Lara Ware, Andrew R. Gray, Alex Chisholm, Siew Ling Tey

**Affiliations:** 1 Department of Human Nutrition, University of Otago, P.O. Box 56, Dunedin 9054, New Zealand; lara.ware@otago.ac.nz (L.W.); alex.chisholm@netspeed.net.nz (A.C.); siewling.tey@otago.ac.nz (S.L.T.); 2 Biostatistics Centre, Division of Health Sciences, University of Otago, P.O. Box 56, Dunedin 9054, New Zealand; andrew.gray@otago.ac.nz

**Keywords:** postprandial glycaemic response, almonds, nuts, satiety, appetite, energy intake, snack foods

## Abstract

Consuming nuts may have advantages over other snack foods for health and body-weight regulation. Suggested mechanisms include increased satiety and lower glycaemia. We used an acute randomised crossover trial to assess glycaemic and appetite responses to consuming two isocaloric snacks (providing 10% of participants’ total energy requirements or 1030 kJ (equivalent to 42.5 g almonds), whichever provided greater energy): raw almonds and sweet biscuits among 100 participants with available data (25 males and 75 females) following 106 being randomised. Two hours after consuming a standardised breakfast, participants consumed the snack food. Finger-prick blood samples measuring blood glucose and subjective appetite ratings using visual analogue scales were taken at baseline and at 15 or 30 min intervals after consumption. Two hours after snack consumption, an ad libitum lunch was offered to participants and consumption was recorded. Participants also recorded food intake for the remainder of the day. The mean area under the blood glucose response curve was statistically and practically significantly lower for almonds than biscuits (mean (95% CI) difference: 53 mmol/L.min (45, 61), *p* < 0.001). Only the composite appetite score at 90 min was higher in the almond treatment compared to the biscuit treatment (45.7 mm vs. 42.4 mm, *p* = 0.035 without adjustment for multiple comparisons). There was no evidence of differences between the snacks for all other appetite ratings or for energy intake at the ad libitum lunch. However, mean energy intakes following snack consumption were significantly lower, both statistically and in practical terms, for the almond treatment compared to the biscuit (mean (95% CI) diff: 638 kJ (44, 1233), *p* = 0.035). Replacing popular snacks with almonds may have advantages in terms of glycaemia and energy balance.

## 1. Introduction

Nuts are more often consumed as snacks rather than meals [1,2]. Given that snacking is common, and has reportedly increased in recent years [3], encouraging more healthful snack options is likely to support energy balance and positively impact diet quality. Nuts provide a more nutrient-dense alternative to some commonly consumed snack foods. Recently, Rehm et al. used food-pattern modelling to assess the impact of substituting typical snacks with tree nuts or almonds only [4]. Substitution using either nuts or almonds improved diet quality, in particular, increasing unsaturated fat, fibre, magnesium and potassium, while decreasing saturated fat and sodium. Furthermore, Tey et al. reported improvements in overall diet quality among free-living participants provided with daily nuts to consume rather than other snack foods [5].

In addition to their nutrient-rich content, nuts may provide additional benefits in terms of energy balance. Cross-sectional studies have observed that nut consumers are leaner than non-nut consumers [6,7,8,9,10,11], and several intervention studies have found no evidence of weight gain in the short-term following the addition of nuts to the diet [5,12,13,14,15]. Metabolic mechanisms suggested for this lack of weight gain include a higher metabolic rate due to the high unsaturated fat content of nuts, and reduced bioaccessibility of lipids because the cell wall of intact nuts limits the release of lipids, resulting in loss of metabolisable energy due to lower fat absorption and higher faecal fat loss. A further mechanism is increased satiety, which includes a behavioural component. Satiety occurs in response to a number of signals including sensory, cognitive and other post-prandial physiological processes such as gastrointestinal peptide release [16]. Foods invoking greater satiety may be useful in body-weight regulation [16]. They have the potential to displace other foods, and this may ultimately influence food choice. Nuts are high in fibre and protein—nutrients, which may promote satiety. Satiety may be further enhanced by the crunchy textural property of whole nuts as the mechanical effort required for mastication with long oral residence time results in the cephalic response and secretion of appetitive hormones [17,18,19,20,21,22,23].

A number of studies have shown that nut consumption results in better appetite ratings and/or reduced subsequent food intake compared to other foods, although this finding is not consistent [24]. A recent meta-analysis found that nut consumption was associated with a reduction in hunger but had no effect on fullness [24]. It should be noted that there was a high level of heterogeneity for each outcome. There is some evidence that nuts eaten as snacks suppress appetite more than when eaten as part of a meal [25]. Tan et al. fed almonds as part of breakfast, lunch or as morning or afternoon snacks. Ratings of hunger and the desire to consume were reduced to a greater extent when the nuts were consumed as snacks [26].

Nuts may increase satiety by eliciting a more stable glycaemic response. Research has suggested that glycaemia may influence satiety, although this finding is not consistent across all studies [27,28,29]. Some short-term studies have shown that in comparison to foods eliciting a higher blood glucose response, lower-glycaemic-index foods may delay subsequent hunger and hence reduce energy intakes [30]. A review examining the acute effects of nut consumption on glycaemia showed that most studies in which nuts were added to meals or were eaten as snacks reduced postprandial glycaemia among healthy participants, as well as those with impaired glucose tolerance and type 2 diabetes [31]. This review also suggested this effect persisted, as longer-term studies produced similar results. In agreement with this, a meta-analysis of 12 intervention studies with interventions ranging from 4 weeks to 12 months showed the consumption of tree nuts (median dose 56 g/d) was associated with significant reductions in glycated haemoglobin (HbAlc) and fasting blood glucose among those with type 2 diabetes [32]. A further meta-analysis of 40 clinical trials with a median intervention period of 3 months (median dose 52 g/d) reported improved insulin sensitivity, but no effect on fasting glucose or HbA1c among participants with and without type 2 diabetes. [33].

Studies that have examined the acute appetite and/or glycaemic responses to nuts have had relatively small samples [34,35], only included females [36,37], or not directly compared outcomes to an isocaloric control snack food [26]. Adequately powered and well-conducted studies with an appropriate comparison group are important for formulating evidence-informed guidelines regarding nut consumption. Investigating factors that influence body weight is particularly important given that research from a number of different groups has consistently shown that a barrier to eating nuts is the fear of weight gain [38,39,40]. We aimed to compare the acute effects of consuming almonds vs. a popular isocaloric snack food (sweet biscuits) on appetite ratings and postprandial glucose responses. We also assessed whether consumption of these snacks influenced subsequent food intake and food choice in the short-term. Sweet biscuits were chosen as the comparator because they are one of the most commonly consumed snack foods in New Zealand [41].

## 2. Materials and Methods

### 2.1. Study Design

This was an acute randomised crossover study involving two treatments. The treatments comprised at least 10% of energy from either raw almonds or sweet biscuits. Participants were randomly allocated to the intervention so that they received the two treatments in a balanced order. The randomisation order was generated using a computerised random-number generator. There was at least a one-week washout between treatments, which was expected to be sufficient for key outcomes to return to pre-intervention levels [42,43].

### 2.2. Participants

Participants were recruited from Dunedin, New Zealand. Recruitment involved the distribution of flyers around supermarkets, gyms, the university campus and the public library. Prospective participants contacted the research team by phone or email. They were asked to complete an online recruitment questionnaire, which included questions related to demographics, physical activity, current snacking habits and health information relevant to study outcomes. Participants could choose multiple options for ethnicity and they were categorised based on the prioritisation order: Māori, Pacific, Asian, Other and European. Participants were asked to report their gender as free text.

Inclusion criteria were healthy men and women aged 18 to 65 years with a body mass index (BMI) 18.5 to 29.9 kg/m^2^ who reported typically consuming at least 1500 kJ of discretionary foods each day. Participants were excluded for the following reasons: if they were on a weight loss/gain diet; had dentition issues preventing the consumption of nuts; had allergies to nuts, wheat or dairy; smoked cigarettes; took supplements containing vitamin E (vitamin E was an outcome in the subsequent long-term study, which participants could take part in); were pregnant or lactating; or had been diagnosed with any chronic illness including diabetes. Participants provided informed written consent and the protocol was approved by the University of Otago Human Ethics Committee (Health), reference number H18/109. The study was registered at the Australian New Zealand Clinical Trials Registry (https://www.anzctr.org.au (accessed on 8 June 2021)), registration number ACTRN12618001758291.

### 2.3. Study Protocol

**Figure 1:**
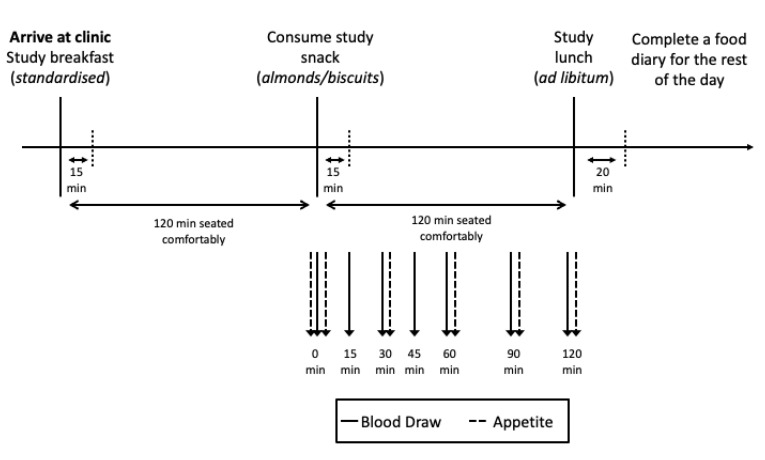
Study protocol.

Participants attended an initial clinic appointment to confirm that they were eligible. In particular, height and weight were assessed to ensure they were within the BMI criteria of 18.5 to 29.9 kg/m^2^. Height was measured to the nearest millimetre using a stadiometer (Holtain Ltd., Crymych, UK), and weight was measured to the nearest 0.1 kg on electronic scales (Seca Alpha 770, Seca, Birmingham, UK). Eligible participants were then scheduled for the two intervention visits. Participants were asked to choose a time between 0700 and 0930 to arrive at the clinic, which would be close to when they usually consumed breakfast, and to arrive at the same time and on the same day of the week for both sessions. They were asked to fast for at least 10 h prior to arriving at the clinic, and to refrain from alcohol consumption and any unusual exercise and activity the day before each session. They were also asked to consume a similar evening meal the night prior to each test. These pre-test requirements were confirmed by a questionnaire completed by participants on each test morning and checked by a researcher. For each session, participants were provided with a standardised breakfast upon arrival at the clinic. They were asked to consume this over a 15 min period. This was followed by a 2-h period where participants were seated comfortably. Two hours following breakfast, participants were provided with their allocated snack of either almonds or sweet biscuits. Glycaemic response and appetite ratings to these snack foods were measured over the following 2 h, where again participants were seated comfortably. Blood glucose was measured at baseline and 15, 30, 45, 60, 90 and 120 min after beginning to eat. Subjective appetite ratings were recorded at baseline, then immediately post-consumption, and at 30, 60, 90 and 120 min post-consumption. At the end of the two hours, participants were offered an ad libitum lunch where their intake was recorded. For the remainder of the day, participants were asked to record all food and drink consumed using paper diaries (Figure 1).

### 2.4. Preload Breakfast

Participants began each acute session by consuming a standardised breakfast. The standard breakfast consisted of a single serving of ready-to-eat muesli (Toasted Muesli Golden Oats and Fruit; Sanitarium, Auckland, Christchurch, New Zealand), plain unsweetened low-fat yoghurt (Dewinkel Plain Unsweetened Yoghurt, Fonterra Brands Ltd., Auckland, New Zealand) and 0.1% fat milk (Anchor Trim Milk, Fonterra Brands Ltd., Auckland, New Zealand). Each participant received a portion equivalent to approximately ~20% (mean (standard deviation (SD)) 19.5% (0.3)) of their total energy requirement (TER). The standard breakfast contained an average of 50% energy from carbohydrates, 16% energy from protein and 30% energy from fat. TER was calculated using the FAO/WHO/UNU equation [44]. This figure was also used to calculate the amount of the test snack provided.

### 2.5. Test Snack Foods

The test foods were raw almonds (Almond Board of California, Modesto, CA, USA) or sweet biscuits (Griffin’s Chocolate Chippie and Chocolate Fingers (Griffin’s Food Company, Manukau City, New Zealand) and Arnott’s Nice, Tiny Teddy and Chocolate Butternut Snap (Arnotts, Pty, Ltd., Silverwater, Australia), and comprised the highest of 10% of TER or the energy equivalent of 42.5 g of almonds (1030 kJ). Sweet biscuits were chosen as the control as these were shown to be one of the most commonly consumed snack foods in the latest adult national nutrition survey in New Zealand [41]. Data from the 2008/09 New Zealand Adult Nutrition Survey was used to determine the most commonly consumed biscuit varieties in New Zealand [45]. The nutritional composition of the test snack foods is shown in Table 1.

### 2.6. Glycaemic Response Protocol

To test the glycaemic response to the test snack foods, participants firstly provided two baseline finger-prick blood samples using Unistik^®^ 3 Normal single-use lancets (Owen Mumford Ltd., Oxford, UK). These capillary samples were immediately analysed for blood glucose using a HemoCue^®^ Glucose 201 Analyser (Helsingborg, Sweden). After these baseline blood samples were taken, participants consumed their allocated snack along with 250 mL water. They were asked to consume this at a comfortable pace over a 15 min period. A further 6 capillary blood samples were taken at 15, 30, 45, 60, 90 and 120 min after beginning to consume the allocated snack.

On the morning of each testing session, the HemoCue^®^ Glucose 201 Analyser (Helsingborg, Sweden) was tested for quality control by the researcher. Each participant’s two testing sessions were separated by at least one week, with a maximum of seven weeks.

Incremental area under the curve (iAUC) for blood glucose was calculated using the trapezoidal rule [43], where negative values were treated as zero.

### 2.7. Satiety Testing Protocol

During each testing session, participants were asked to complete an appetite-rating questionnaire at six time points: at baseline before the consumption of each test snack food, then immediately post-consumption, and at 30, 60, 90 and 120 min post-consumption. The questionnaire comprised 100 mm visual analogue scales with five questions at each time point on hunger, fullness, desire to eat, prospective consumption and preoccupation with thoughts of food [42].

It has been suggested that combining ratings into an appetite score may be a more robust measure of appetite [42]. We calculated an appetite score for each time point by calculating the arithmetic mean for scores for hunger, desire to eat, prospective food consumption, preoccupation with thoughts of food and the reversed score for fullness. A low score corresponds to a low level of appetite. The total area under the curve for each appetite rating and the appetite score was calculated.

We also calculated a satiety quotient (SQ) for each of the five appetite ratings at each time point following consumption of the snacks. This is calculated using the following formula:

$$\mathrm{Satiety}\;\mathrm{quotient}\;(\mathrm{mm}/\mathrm{kJ})=\frac{\mathrm{rating}\;\mathrm{pre}-\mathrm{consumption}\;\mathrm{of}\;\mathrm{snack}\;(\mathrm{mm})-\mathrm{rating}\;\mathrm{post}-\mathrm{consumption}\;\mathrm{of}\;\mathrm{snack}\;(\mathrm{mm})}{\mathrm{Energy}\;\mathrm{intake}\;\mathrm{of}\;\mathrm{the}\;\mathrm{snack}\;\mathrm{food}\;(\mathrm{kJ})}\times 100$$

Rating refers to the appetite measure from the visual analogue scale. This provides some understanding of the extent to which the snack food eaten reduces the subjective rating (e.g., hunger) per kJ. A higher SQ, for all ratings except for fullness, represents higher satiety capacity. A higher SQ for fullness represents weaker satiety capacity. The satiety quotient for the almonds was calculated using the Atwater factors (2649 kJ per 100 g) and digestible energy values (2013 kJ per 100 g) [46]. We also calculated a mean SQ for each appetite index. This was carried out by calculating the mean of the five post-consumption appetite ratings for that index and using this value in the equation above.

### 2.8. Ad Libitum Lunch

Following the glycaemic index testing protocol, each participant was invited to consume an ad libitum lunch. Each participant was presented with a plate of sandwiches (320 to 330 g) and asked to eat until they were comfortably full, up to 20 min. They were not told that their lunch intake would be measured and assured any uneaten food would not be wasted. Sandwiches were cut unevenly in different sizes to minimise the effect of portion size on intake. Further to this, upon nearing completion of a serving of sandwiches, the plate was replaced with a new portion before the current serving had been eaten in full. Thus, the participants were not given the visual cues of a larger portion that may promote overconsumption, but likewise never had the opportunity to make a decision to end their meal based on an empty plate. The amount of food eaten was calculated by subtracting leftovers from the amount presented. They were asked to record their food intake for the remainder of the test day after leaving the clinic using a paper diary as described below.

### 2.9. Dietary Assessment

Weighed records of all foods and beverages eaten for the remainder of the test days were collected from participants. Participants were not provided with any dietary substitution instructions. Participants were provided with electronic kitchen scales (Salter Electronic, Salter Housewares Ltd., Kent, UK), which were accurate to ±1 g. Detailed verbal and written instructions on how to complete the food record were provided. The records were reviewed by the researcher at the time of collection. All records were analysed by the same researcher for nutrient content using FoodWorks version 9 (Xyris Software, Brisbane, Australia), which uses the New Zealand food composition data NZ FOODfiles 2016 (Plant and Food Research, Palmerston North, New Zealand).

### 2.10. Sample Size and Statistical Analysis

This was designed as a sub-study within a larger longitudinal study where the sample size was based on body weight change over a 1 y intervention. For the larger study, in order to provide 80% power to detect a 1.6 kg difference in weight change over 12 months between the same two snack groups as used here, assuming a SD for weight change of 2.7 kg (estimated from Diaz et al. [47]) and using a two-sided test at the 0.05 level, 46 participants would be required in each group at follow-up (*n* = 92 in total at follow-up). Participants recruited for the present acute study were planned to then take part in this long-term study and so the sample size here was based on recruiting 120 participants so that with a 75–80% retention rate, we would have around 92 participants at the completion of the longitudinal study, although logistics affected these plans and led to the two samples overlapping but not being identical.

Statistical analyses involved linear mixed models with a random effect for participants to accommodate for the crossover design. Comparisons between groups were made without adjustment for multiple comparisons; therefore, care is needed when interpreting findings. The models included the intervention period (first or second) to adjust for any influence of this on outcomes. The effectiveness of the washout period was assessed by visual inspection of the data to identify any evidence for carryover effects. Mean outcomes are presented alongside their associated confidence intervals. For comparison of the appetite score over time, we controlled for baseline scores. Log-transformations were made where this improved residual normality and/or homoscedasticity. These variables are presented as geometric means and associated SDs and confidence intervals. Analyses were performed using Stata 12 with two-sided 95% confidence intervals presented and two-sided tests performed at the 0.05 level.

## 3. Results

### 3.1. Participants

**Figure 2:**
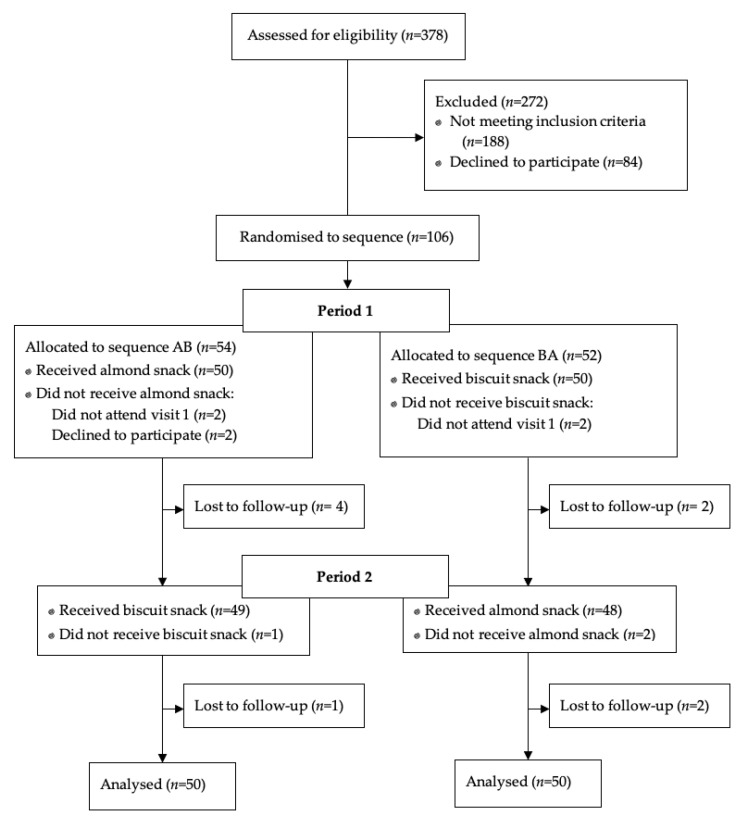
Flow of participants through study.

In total, 378 participants were assessed for eligibility (Figure 2). Of the 190 who were eligible, 106 were recruited and randomised. Six participants did not provide sufficient data for the planned analyses and so the results presented here are based on 100 participants. The characteristics of participants are presented in Table 2. There was a higher proportion of females and NZ Europeans. The median age was 29 years with a range from 18 to 65 years.

### 3.2. Glycaemic Response

**Figure 3:**
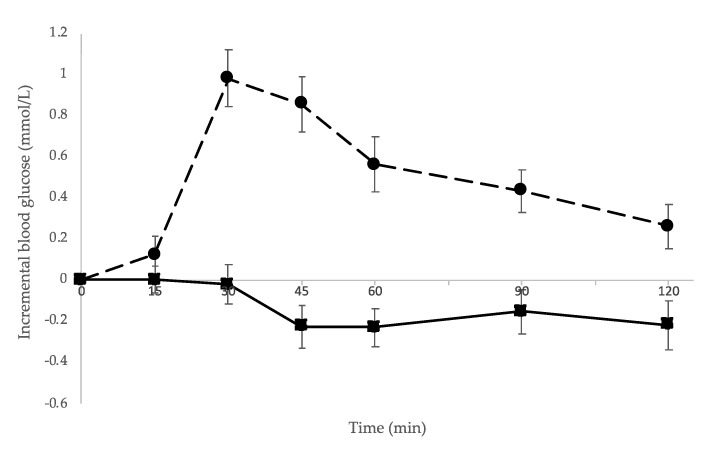
Mean (95% CI) incremental blood glucose response after consuming almonds and biscuits. iAUC was compared using a linear mixed model with random effects included for participants to accommodate the crossover design. iAUC (calculated using the trapezoidal rule [43], where negative values were treated as zero) for the almonds was statistically and practically significantly lower compared to the biscuits (*p* < 0.001). Solid line with ■, almonds; dashed line with ●, biscuits.

Figure 3 presents the mean incremental blood glucose responses after consuming the almonds and the biscuits as a mid-morning snack. The mean (95% CI) difference in incremental blood glucose area under the curve was 53 mmol/L.min (45, 61), (*p* < 0.001).

### 3.3. Appetite Ratings

#### 3.3.1. Area under the Curve for Appetite

There was no statistically or practically significant evidence for differences in AUC for any of the appetite ratings or for the appetite score between the almond and biscuit snacks (all *p* ≥ 0.097) (Table 3).

#### 3.3.2. Appetite Scores

After controlling for baseline values, only the appetite score at 90 min was statistically significantly different between treatments (unadjusted *p* = 0.035), with a lower score for the biscuits compared to the almonds (Table 3). A lower score indicates a lower level of appetite. There was no statistically significant evidence for differences between the snacks for appetite score at any of the other time points (all *p* ≥ 0.109).

#### 3.3.3. Satiety Quotient

A higher SQ for all ratings except for fullness indicates higher satiety capacity. A higher SQ for fullness represents weaker satiety capacity. The mean SQ for hunger when using the Atwater factors for energy was statistically significantly lower for the almonds compared to biscuits (*p* = 0.037) (Table 3). This difference was no longer statistically significant when digestible energy was used in the equation. There was no other statistically significant evidence for differences in the mean SQ for the other appetite measures when using the Atwater factor energy or digestible energy.

**Figure 4:**
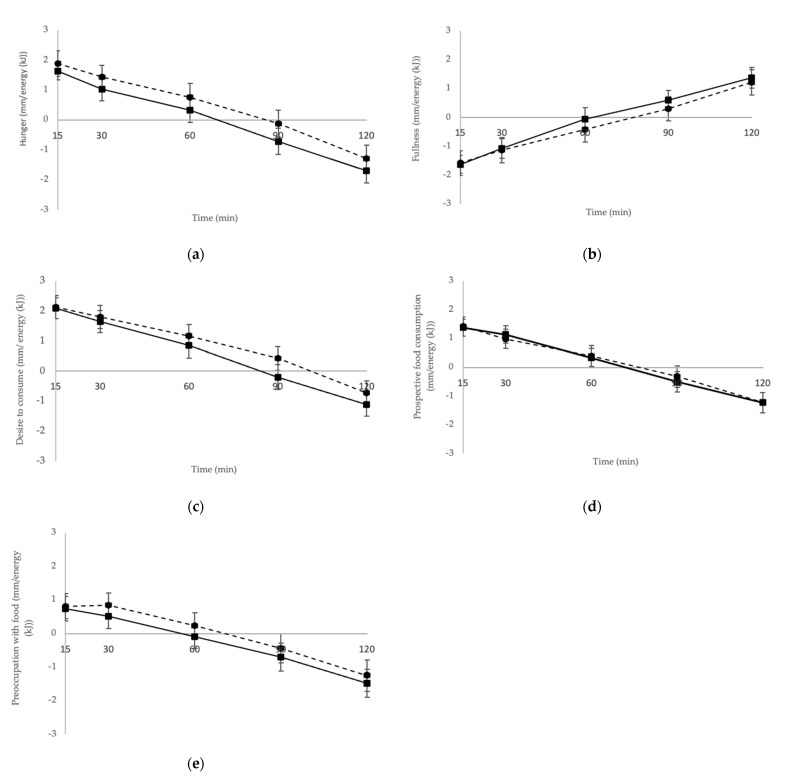
Mean (95% CI) satiety quotient (using Atwater factor energy) immediately after consuming the snack to 120 min for: (**a**) Hunger; (**b**) fullness; (**c**) desire to consume; (**d**) prospective food consumption; (**e**) preoccupation with food. Solid line with ■, almonds; dashed line with ●, biscuits.

Plotting the SQ over time using Atwater energy shows that the SQ for almonds was lower for most time points compared to the biscuits for hunger, desire to consume and preoccupation with food (Figure 4). The SQ for fullness and prospective food intake were similar between the two treatments over time.

Plotting SQ over time using digestible energy shows that SQ was similar until around 60 min, whereafter it was lower for almonds (Figure S1). The SQ for the desire to consume and prospective food consumption was higher for the almonds over the first 60 min, after which it was lower compared to the biscuits. The SQ for preoccupation with food was similar in both treatments to around 30 min, after which it is higher for the biscuits. The SQ for fullness was lower for almonds at 30 min, but thereafter was higher compared to biscuits.

### 3.4. Energy and Nutrient Intakes

**Figure 5:**
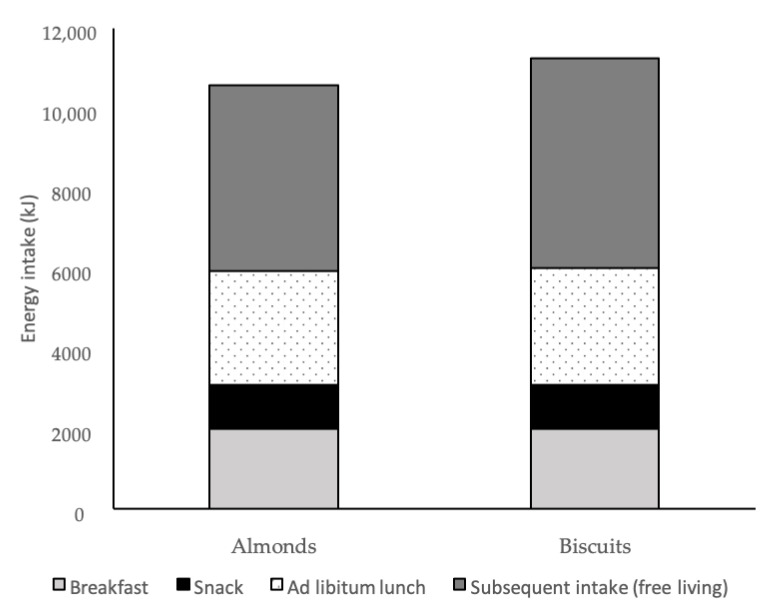
Mean energy intakes consumed during the standard breakfast (light grey bars), the standardised morning study snack (black bars), the ad libitum lunch (dotted bars) and subsequent intake for the remainder of the day (free living) (dark grey bars).

Energy intakes from the different stages of the study are shown in Figure 5. The mean (SD) energy intake from the snack foods was 1090 (109) kJ for almonds and 1094 (112) kJ for biscuits. The mean (SD) energy intake from the standardised breakfast was 2006 (344) kJ.

The mean (SD) intake at the ad libitum lunch was 2820 (1343) kJ for almonds and 2898 (1329) kJ for biscuits. The mean (95% CI) difference in energy intake between the two treatments was 96 kJ (−60, 252), (*p* = 0.226) (Figure 4). The mean (SD) energy intakes from food diaries participants completed over the remainder of the day were 4658 (2517) kJ and 5200 (2676) kJ for almond and biscuit treatments, respectively. The mean (95% CI) difference between the two treatments for the remainder of the day were 542 kJ (−40, 1124), *p* = 0.068).

When energy intakes from the ad libitum lunch and for the remainder of the day were combined, mean (95% CI) energy intakes were 638 kJ (44, 1233) (*p* = 0.035) lower after consumption of the almond snack compared to the biscuit snack (Table 4 and Figure 3). There were no statistically significant differences in other nutrients, although there was a non-statistically significant tendency for lower absolute intakes of saturated fat (*p* = 0.056) and sugar (*p* = 0.053) in the almond treatment compared to the biscuit treatment.

## 4. Discussion

We found that snacking on almonds resulted in a statistically and practically significantly lower glycaemic response compared to snacking on sweet biscuits. Although this did not translate into evidence for differences in appetite ratings, statistically significantly less energy was consumed over the testing day when the mid-morning snack comprised almonds rather than sweet biscuits, suggesting a practically important (approximating more than half a kilo weight gain/loss per month) effect of almonds relative to biscuits.

Previous studies have shown that participants compensate for a substantial proportion of energy consumed from nuts when they are added to the diet. Interestingly, a recent meta-analysis showed that the lack of weight change was observed whether or not they received dietary substitution instructions [48]. Hull et al. found that energy intake at lunch and dinner following a mid-morning snack of 28 g or 42 g of almonds decreased in a dose-dependent manner [37]. However, twenty-four-hour energy intake did not statistically significantly differ between the two nut doses and a no-nut control, suggesting energy compensation took place in the nut treatments. We showed practically important lower intakes over a 24-h period when the mid-morning snack was comprised of almonds compared to an isocaloric biscuit snack. Barbour et al. reported similar results to our study both in terms of energy intakes and satiety ratings [49]. They found no evidence for differences in perceived satiety ratings over a three-hour period after consuming isocaloric amounts of peanuts (56–84 g), hi-oleic peanuts (56–84 g) or potato crisps (60–90 g). However, they did find statistically and practically significantly lower energy intakes at an ad libitum lunch when participants consumed peanuts (−17%) and hi-oleic peanuts (−21%), and this lower energy intake persisted over 4 days of consumption of these snacks. Other studies have not shown evidence for differences in energy intakes. Although Hollingworth et al. noted beneficial differences in appetite ratings, particularly lower desire-to-eat scores, among women consuming a mid-morning snack of almonds compared to those consuming crackers, they did not observe evidence for differences in 24 h intakes between the two snack foods [36]. A meta-analysis by Akhlaghi found, in a subgroup analysis, evidence that eating nuts increased energy intake in overweight and obese individuals, but not in those with a normal BMI [24]. Our study comprised healthy and overweight males and females and excluded obese individuals. The inclusion of participants from different BMI categories may account for some of the mixed findings in this area, and future studies should examine the relationship between nut consumption and energy intakes among groups of different weight categories.

A number of properties of nuts such as fibre and protein content and the crunchy texture may contribute to the lack of overconsumption in comparison to other popular snack foods. These factors may influence perceived satiety, oral exposure time and reduced post-prandial drive for food intake [25]. 

Nutrient analysis of the diet for the remainder of the test day showed that there was a non-statistically significant tendency for lower absolute intakes of saturated fat and sugar after the almond treatment compared to the biscuit treatment. This suggests that consuming almonds as a snack may improve overall diet quality compared to other popular snack foods. This analysis did not include the study snack foods themselves. Given the nutrient profile of almonds, which are rich sources of unsaturated fats, vitamins and minerals, the difference in nutrient quality would be even more pronounced after their inclusion. In a longer trial over three months, Tey et al. reported that nut consumption improved overall diet quality, in particular reducing saturated fat and increasing monounsaturated and polyunsaturated fat compared to other popular snack foods, namely chocolate and potato crisps [5]. 

The lower blood glucose response to almonds compared to biscuits is not unexpected given the lower glycaemic carbohydrate content of nuts compared to biscuits. Other studies have shown improved acute glycaemic control when nuts are consumed as snacks compared to other snack foods such as savoury crackers [35,36]. In addition, when nuts have been added to high-carbohydrate foods such as bread or muffins, post-prandial glycaemia has decreased [34,50,51,52]. Interestingly, in our study, the lower post-prandial response elicited by almonds compared to biscuits was not reflected in changes in appetite ratings. Although the lower glycaemic response was not associated with appetite scores, this physiological response to almonds is likely to have a number of health benefits. Increased postprandial glycaemia is thought to be associated with cardiovascular disease and total mortality among those with and without type 2 diabetes [28]. Studies of longer duration indicate that the effects of nuts on lowering glycaemia persist [31,53,54]. 

The lack of evidence for differences in appetite ratings between the two treatments is interesting given the statistically and clinically significantly lower postprandial glycaemic responses and 24 h energy intakes with the almond treatment compared to the biscuit treatment. The mean differences in AUC for appetite ratings were in a range of 2–8%. Blundell et al. have suggested that a mean difference in response of 8–10% is practically relevant [42]. Previous research has shown mixed results in terms of appetite ratings. Unlike a meta-analysis that found that nut consumption was associated with lower hunger ratings but not fullness ratings, our study found a lack of evidence that appetite ratings differed meaningfully between treatments [24]. In fact, our study showed that the satiety quotient for hunger was statistically significantly higher for the biscuit treatment. A higher satiety quotient for hunger indicates a higher suppression of hunger per kJ of the snack. This difference was no longer statistically significant when we used digestible energy for the nuts. Given the fact we did not adjust for multiple comparisons, the statistically significant difference (*p* = 0.037) needs to be interpreted with caution until this finding can be replicated. It has been suggested that the satiety quotient may provide different information than appetite ratings alone. A recent systematic review suggested that the satiety quotient could be used as a predictor of energy intake. Other studies have also shown negative associations between satiety quotient and body weight, fat mass and waist circumference, and positive associations between the satiety quotient and weight loss [55]. The timeline of the satiety quotient can also clarify whether the more immediate post-absorptive effects on appetite ratings differ from the effects produced later. This is important in order to differentiate foods that may be suppressing appetite in the short term, compared to foods that may result in more prolonged suppression. In our study, for many of the ratings, almonds appeared to suppress appetite ratings more immediately after eating, whereas long-term suppression was greater with the biscuits. However, our ratings were only assessed over a 2 h period and it would be interesting to observe these for longer given that 24 h energy intakes were lower after the almond treatment. Other acute studies have reported similar findings to ours. Godwin et al. compared the consumption of mixed nuts and pretzels on blood glucose and appetite ratings over 2 h. Both blood glucose concentrations and satiety ratings were higher after pretzel consumption compared to the mixed nuts [56]. Conversely, Mori et al. observed that the inclusion of whole almonds as part of a breakfast meal increased satiety and attenuated blood glucose response after a second meal consumed 4 h after breakfast among participants with impaired glucose tolerance [57]. Over 24 weeks, Wang et al. showed that the inclusion of mixed nuts (1.5 oz/d) into a weight loss diet increased satiety to a greater extent than isocaloric amounts of pretzels [58]. However, it should be noted that there was no evidence that weight loss differed between the two groups. 

There are a number of limitations that should be considered when interpreting the results of the current study. First, the acute nature of the study provides useful information over the short-term, but the results will not necessarily translate into long-term behaviour. We only measured appetite ratings during the 2 h following consumption of the snacks. Given the lower 24 h energy intake among the almond group, it would be useful to measure appetite ratings for longer periods. However, we speculate such monitoring in itself may influence eating behaviour and food intake. We performed multiple between-group comparisons in the statistical analyses, which increase the type 1 error rate, and the possibility that some of the statistically significant findings were due to chance should be kept in mind until they can be replicated. Because of the different nutrient compositions of the snack foods, we were unable to control for energy density. We only examined blood glucose concentrations over a 2 h period following snack consumption. Monitoring blood glucose concentrations over longer periods may have been more informative given the fact research has shown that whole almonds reduced glycaemia acutely and effects persisted after a second meal [57], although this would have increased participant burden in this study. The use of technologies such as the continuous glucose-monitoring system would be useful to investigate this question. We also only included normal weight and overweight participants and have not examined effect modification by weight status given the numbers of participants in each. Obese individuals may respond differently, and so future research could be designed to examine potential differences between weight categories, as well as between groups defined by gender, age, and socioeconomic status.

One strength is our comparison snack was carefully chosen, being the most commonly consumed snack food identified from a national nutrition survey of New Zealanders, increasing external validity. We also had good retention, with 100 of the 106 participants randomised providing analysable data.

## 5. Conclusions

In comparison to sweet biscuits, consuming almonds as a mid-morning snack improved acute glycaemia and reduced energy intake over the remainder of the test day to a practically meaningful extent. There were some non-statistically significant tendencies for improvement in diet quality. Therefore, replacing less healthy snacks with almonds may provide a variety of benefits.

## Figures and Tables

**Table 1:** Nutritional composition of the study snack food expressed per 100 g and as a percent of total energy.

	Raw Almonds *	Biscuits †
*Per 100 g*		
Energy (kJ)	2423	1972
Protein (g)	21.2	5.4
Total fat (g)	49.9	18.2
Carbohydrate (g)	21.6	70.1
*Percent of total energy per serve*		
Protein	14.9	4.7
Total fat	76.2	34.1
Carbohydrate	10.2	60.4

* Sourced from U.S. Department of Agriculture, Agricultural Research Service, Nutrient Data Laboratory. USDA National Nutrient Database for Standard Reference, Release 28. Version Current: September 2015. http://www.ars.usda.gov/nea/bhnrc/ndl (accessed on 28 September 2021); † sources from the nutrient information panels of the products.

**Table 2:** Characteristics of study participants (*n* = 100).

Demographic Variable	All Participants
Age (y) †	29 (21, 41)
Gender	
Male, *n* (%)	25 (25)
Female, *n* (%)	75 (75)
Height (m)	1.69 (0.09)
Weight (kg)	65.95 (11.54)
BMI (kg/m^2^)	23.1 (2.8)
Ethnicity *n* (%)	
NZ European	61 (61)
Māori	9 (9)
Asian	26 (26)
Other	4 (4)

All values are means (SD) unless otherwise specified; † median (25th and 75th percentiles).

**Table 3:** Mean (SD) two-hour area under the curve (AUC) and satiety quotient for each appetite rating, and appetite score over time.

Variable	Almonds	Biscuits	Mean (95%CI) Difference	*p*-Value *
*2-h area under the curve (2-h AUC, mm x min)*				
2-h AUC for hunger	4247 (2353)	3921 (2029)	−310 (−699, 72)	0.119
2-h AUC for fullness	6215 (2139)	6380 (2110)	132 (−252, 515)	0.501
2-h AUC for desire to consume	4515 (2415)	4176 (2088)	−334 (−729, 61)	0.097
2-h AUC for prospective food consumption	4730 (2269)	4602 (1977)	−121 (−497, 255)	0.530
2-h AUC for preoccupied with food	3577 (2567)	3459 (2294)	−118 (−466, 230)	0.506
2-h AUC for appetite score	4572 (2169)	4331 (1899)	−235 (−558, 87)	0.152
*Appetite score over time (mm)*				
Baseline	39.6 (18.4)	40.7 (18.7)		
15 min	23.7 (17.9)	24.0 (17.7)	−0.3 (−3.3, 2.7)	0.823
30 min	28.2 (19.5)	27.5 (17.7)	−1.4 (−4.4, 1.7)	0.382
60 min	36.6 (20.6)	34.4 (18.0)	−2.7 (−6.0, 0.60)	0.109
90 min	45.7 (20.8)	42.4 (19.1)	−3.6 (−6.9, −0.3)	0.035
120 min	54.5 (20.7)	52.8 (18.8)	−1.9 (−5.5, 1.6)	0.282
*Mean post-consumption satiety quotient (SQ, mm/kJ) using Atwater factors*				
SQ for hunger	0.103 (1.655)	0.545 (1.799)	4.336 (0.026, 0.841)	0.037
SQ for fullness	−0.131 (−1.251)	−0.271 (1.494)	−0.135 (−0.492, 0.222)	0.457
SQ for desire to consume	0.539 (1.311)	0.798 (1.375)	0.263 (−0.085, 0.612)	0.139
SQ for prospective food consumption	0.180 (1.122)	0.213 (1.210)	0.026 (−0.241, 0.293)	0.849
SQ for preoccupied with food	−0.176 (1.390)	0.033 1.523)	0.188 (−0.156, 0.531)	0.284
*Mean post-consumption satiety quotient (SQ, mm/kJ) using digestible energy*				
SQ for hunger	0.122 (2.153)	0.545 (1.799)	0.411 (−0.053, 0.876)	0.082
SQ for fullness	−0.173 (1.646)	−0.271 (1.494)	−0.093 (−0.500, 0.314	0.653
SQ for desire to consume	0.713 (1.721)	0.798 (1.375)	0.090 (−0.315, 0.495)	0.663
SQ for prospective food consumption	0.237 (1.476)	0.213 (1.210)	−0.033 (−0.342, 0.273)	0.835
SQ for preoccupied with food	−0.227 (1.825)	0.033 (1.523)	2.35 (−0.160, 0.629)	0.243

* All *p*-values from linear mixed models (comparisons between groups were made without adjustment for multiple comparisons; therefore, care is needed when interpretating the findings as this can increase the type 1 error rate); values are presented as arithmetic means and SD.

**Table 4:** Mean (SD) energy and nutrient intakes for the ad libitum lunch and for the remainder of the testing day combined.

Variable	Almonds	Biscuits	Mean (95%CI) Difference	*p*-Value *
Energy (kJ)	7494 (2589)	8132 (3044)	638 (44, 1233)	0.035
Protein g	78.8 (31.7)	80.1 (29.1)	1.3 (−3.8, 6.5)	0.612
Protein (%TE)	18.1 (4.1)	17.3 (3.8)	−0.9 (−2.0, 0.2)	0.110
Carbohydrate (g) †	185 (1.42)	197 (1.46)	1.06 (0.98, 1.16)	0.150
Carbohydrate (%TE)	44.8 (6.8)	44.5 (7.9)	−0.2 (−1.9, 1.4)	0.771
Total fat (g) †	66.0 (1.5)	70.9 (1.54)	1.07 (0.98, 1.18)	0.125
Total fat (%TE)	35.2 (6.6)	35.2 (7.6)	0.1 (−1.7, 1.8)	0.938
Saturated fat (g)	26.8 (11.6)	29.7 (15.3)	2.9 (−0.1, 5.9)	0.056
Saturated fat (%TE)	13.2 (3.5)	13.4 (4.4)	0.2 (−0.8, 1.1)	0.725
Monounsaturated fat (g) †	21.0 (1.66)	22.5 (1.64)	1.07 (0.95, 1.20)	0.258
Monounsaturated fat (%TE) †	10.9 (1.36)	10.9 (1.40)	0.99 (0.91, 1.08)	0.906
Polyunsaturated fat (g) †	12.3 (1.54)	12.9 (1.54)	1.05 (0.96, 1.15)	0.278
Polyunsaturated fat (%TE)	6.7 (1.8)	6.6 (2.1)	−0.1 (−0.5, 0.3)	0.563
Cholesterol (mg) †	133 (2.02)	129 (1.97)	0.97 (0.82, 1.15)	0.747
Sugar (g) †	56.4 (1.84)	64.8 (1.69)	1.15 (1.00, 1.32)	0.053
Sugar (%TE) †	13.5 (1.65)	14.4 (1.52)	10.7 (0.96, 1.18)	0.207
Fibre (g) †	21.9 (1.46)	23.1 (1.57)	1.05 (0.97, 1.14)	0.192

* All *p*-values from linear mixed models; values are presented as arithmetic means and SDs except for those with † where values are geometric means and SDs, and differences are ratios of the geometric means. For geometric means, one SD above the mean would be the product of the mean and SD and one SD below the mean would be the mean divided by the SD.

## Data Availability

The data presented in this study are available on reasonable request from the corresponding author.

## Supplementary Materials

The following are available online at https://www.mdpi.com/article/10.3390/ijerph182010989/s1, Figure S1: Satiety quotient (using digestible energy) immediately after consuming the snack to 120 min for: (**a**) Hunger; (**b**) fullness; (**c**) desire to consume; (**d**) prospective food consumption; (**e**) preoccupation with food. Solid line with ■, almonds; dashed line with ●, biscuits.
