# Supplementary material for: Snacking on Almonds Lowers Glycaemia and Energy Intake Compared to a Popular High-Carbohydrate Snack Food: An Acute Randomised Crossover Study
Source: Int J Environ Res Public Health. 2021 Oct 19;18(20):10989. doi: 10.3390/ijerph182010989 (PMC8535760; doi:10.3390/ijerph182010989)
Supplement: Supplementary file 1 [file ijerph-18-10989-s001.zip › ijerph-1419377-supplementary.pdf]

International Journal of
Environmental Research and Public Health

MDPI

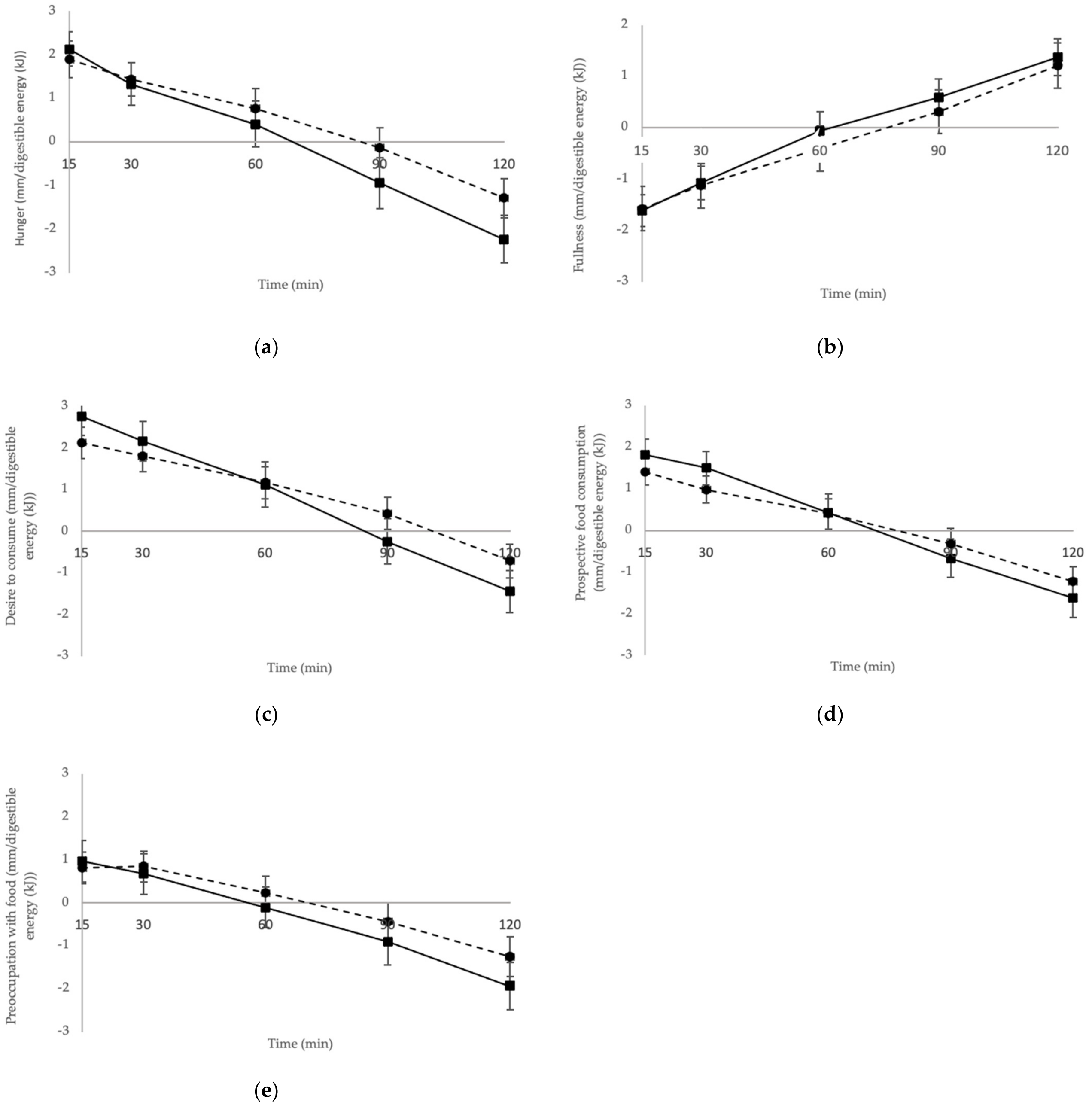

Figure S1. Satiety quotient (using digestible energy) immediately after consuming the snack to 120 minutes for: (**a**) Hunger; (**b**) Fullness; (**c**) Desire to consume; (**d**) Prospective food consumption; (**e**) Preoccupation with food. Solid line with ■, almonds; dashed line with ●, biscuits
